# Live and Let Die - The B_sister_ MADS-Box Gene *OsMADS29* Controls the Degeneration of Cells in Maternal Tissues during Seed Development of Rice (*Oryza sativa*)

**DOI:** 10.1371/journal.pone.0051435

**Published:** 2012-12-12

**Authors:** Xuelian Yang, Feng Wu, Xuelei Lin, Xiaoqiu Du, Kang Chong, Lydia Gramzow, Susanne Schilling, Annette Becker, Günter Theißen, Zheng Meng

**Affiliations:** 1 Key Laboratory of Plant Molecular Physiology, Institute of Botany, Chinese Academy of Sciences, Beijing, People’s Republic of China; 2 Graduate School, Chinese Academy of Sciences, Beijing, People’s Republic of China; 3 Department of Genetics, Friedrich Schiller University Jena, Jena, Germany; 4 Plant Evo-Devo Group, The Institute of Botany, Justus-Liebig-University Gießen, Gießen, Germany; Lawrence Berkeley National Laboratory, United States of America

## Abstract

B_sister_ genes have been identified as the closest relatives of class B floral homeotic genes. Previous studies have shown that B_sister_ genes from eudicots are involved in cell differentiation during ovule and seed development. However, the complete function of B_sister_ genes in eudicots is masked by redundancy with other genes and little is known about the function of B_sister_ genes in monocots, and about the evolution of B_sister_ gene functions. Here we characterize *OsMADS29*, one of three MADS-box B_sister_ genes in rice. Our analyses show that *OsMADS29* is expressed in female reproductive organs including the ovule, ovule vasculature, and the whole seed except for the outer layer cells of the pericarp. Knock-down of *OsMADS29* by double-stranded RNA-mediated interference (RNAi) results in shriveled and/or aborted seeds. Histological analyses of the abnormal seeds at 7 days after pollination (DAP) indicate that the symplastic continuity, including the ovular vascular trace and the nucellar projection, which is the nutrient source for the filial tissue at early development stages, is affected. Moreover, degeneration of all the maternal tissues in the transgenic seeds, including the pericarp, ovular vascular trace, integuments, nucellar epidermis and nucellar projection, is blocked as compared to control plants. Our results suggest that *OsMADS29* has important functions in seed development of rice by regulating cell degeneration of maternal tissues. Our findings provide important insights into the ancestral function of B_sister_ genes.

## Background

Genetic and functional analyses of floral homeotic mutants in the model eudicot plants *Arabidopsis thaliana* and *Antirrhinum majus* led to the formulation of the ABC model, which was proposed to explain the determination of floral organ identities [1]–[2]. According to this model, class A genes specify the identity of sepals, class A and B genes specify petal identity, class B and C genes determine stamen identity, and class C genes determine carpel identity. Most of the floral homeotic genes that play a role in the ABC model are MIKC-type MADS-box genes. These genes were named after the domain structure of the encoded transcription factors consisting of a conserved DNA-binding MADS (M) domain, a less conserved intervening (I) region, a moderately conserved keratin-like (K) domain and a highly variable C-terminal (C) region [3]–[5].

In the ABC model, class B genes specify petal and stamen identity in combination with class A and C genes, respectively [2]. Several years ago, the sister clade of class B genes (*DEF*/*GLO*-like genes, also known as *AP3*/*PI*-like genes), termed B_sister_ genes, was identified in both angiosperms and gymnosperms [6]. Protein sequence alignments with other MADS-domain proteins indicated that compared to other MIKC-type proteins, the proteins encoded by B_sister_ genes share a shorter I domain, a sub-terminal PI Motif-derived sequence and, in some cases, also a PaleoAP3 Motif in the C-terminal region with the AP3/PI-like proteins of gymnosperms and angiosperms [6].

In contrast to class B genes, which are predominantly expressed in male reproductive organs (and in angiosperm petals), B_sister_ genes were found to be mainly transcribed in female reproductive organs (ovules, carpel walls) and in developing seeds [7]–[9]. Furthermore, the sequences of B_sister_ genes are highly conserved. Together these findings suggest that B_sister_ genes play an important role in ovule and/or seed development, which has been conserved for about 300 million years [6].

In *Arabidopsis*, two B_sister_ genes, *ARABIDOPSIS BSISTER* (*ABS*; also known as *TRANSPARENT TESTA 16* (*TT16*) and *AGL32*) and *GORDITA (GOA,* formerly known as *AGL63*), were identified [9]–[12]. Expression analyses showed that *ABS* is expressed in the endothelial layer of the inner integuments of mature ovules [13]. In line with this, the *abs* (*tt16*) mutant seeds showed a loss of pigmentation and defect inner integument, indicating that *ABS* (*TT16*) regulates anthocyanidin accumulation and inner endothelial cell differentiation in *Arabidopsis* [9], [12]. Recently it was shown that *ABS* acts redundantly with the class D floral homeotic gene *SEEDSTICK* (*STK*); *abs*/*stk* double mutants are characterized by a total absence of the endothelium and by massive starch accumulation in the embryo sac resulting in a low number of viable seeds [13].

The closest relative of *ABS* in *Arabidopsis* is *GOA*. *GOA* has a broader expression pattern compared to *ABS*. It has undergone neofunctionalization and has non-redundant functions to *ABS* in ovule outer integument development and the regulation of fruit longitudinal growth [10], [11]. In *Petunia*, the *FLORAL BINDING PROTEIN 24* gene (*FBP24)* is expressed in ovule primodia, nucelli and integuments. Later, its expression is restricted to the endothelium of mature ovules and seeds. An *fbp24* knock-out mutant did not show alterations in development, indicating that *FBP24* acts redundantly with other genes. *fbp24* knock-down lines in which so far unknown other genes are likely co-suppressed, are affected in endothelium layer development similar to *abs* mutants, which suggests that the *FBP24* function is similar to that of *ABS* in *Arabidopsis* [8].

**Figure 1:**
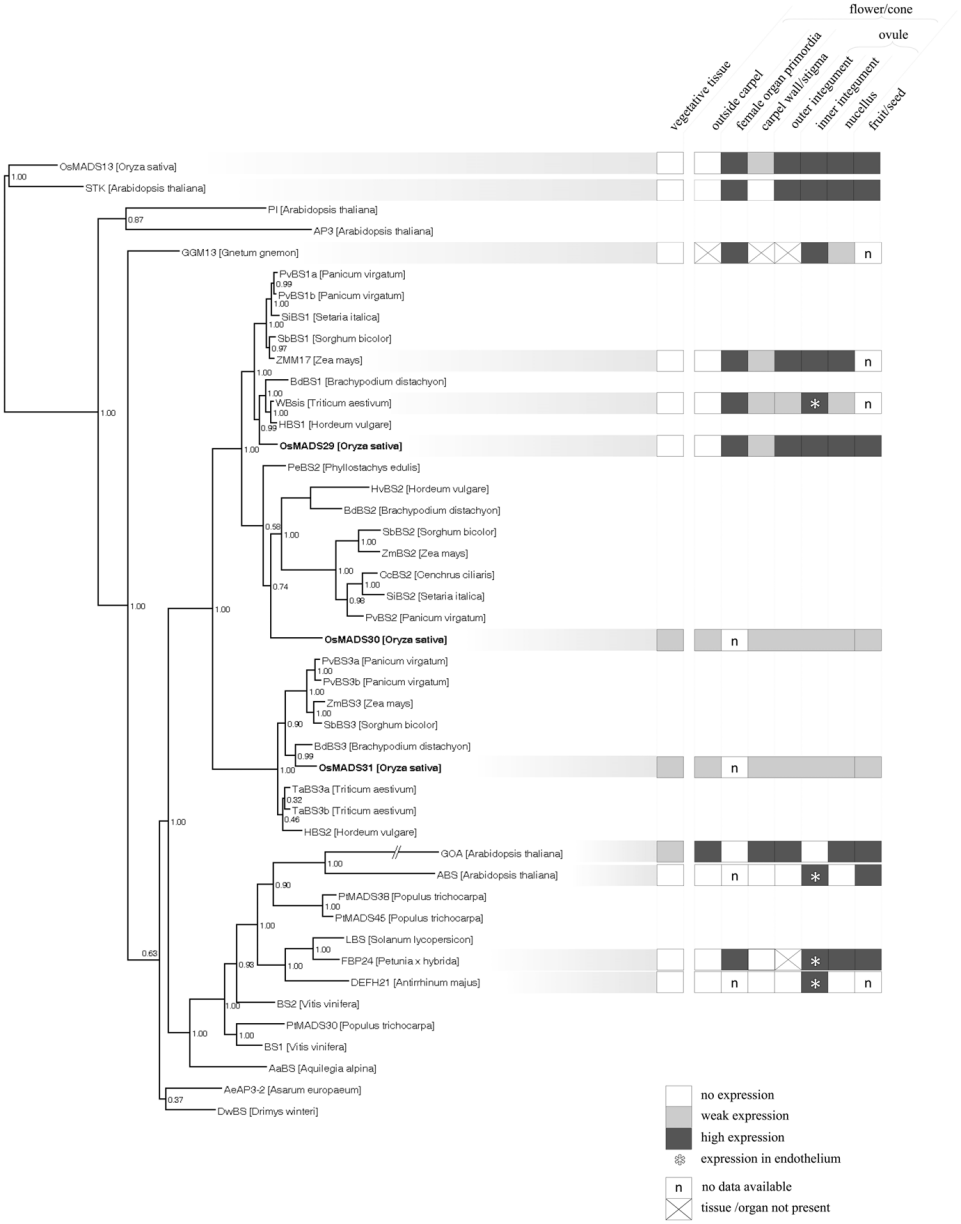
Phylogeny and expression patterns of B_sister_ genes. Bayesian phylogeny of class D, class B and B_sister_ genes in seed plants. Posterior probabilities are indicated on the nodes. The expression patterns of B_sister_ and class D genes are depicted as far as they are known. Colors indicate expression intensity: white, no expression; light grey, weak expression; dark grey, strong expression; asterisk, expression was detected particularly in endothelial cells of inner integument; n, no expression data available; cross, tissue or organ is not present in respective species.

So far, functional studies have been conducted for B_sister_ genes from core eudicots only. However, expression patterns have been determined also for B_sister_ genes from a number of other species, as summarized in Figure 1. The B_sister_ gene *GGM13* of the gymnosperm *Gnetum gnemon* is expressed in the developing nucellus and inner envelope of female reproductive units [6]. Expression analyses of B_sister_ genes in monocots were carried out in several species. The wheat B_sister_ gene, *WBsis*, is expressed in the endothelial layer of the inner integument of the ovule [7], resembling the expression pattern of *ABS* in *Arabidopsis* [13]. However, weak expression of *WBsis* was detected also in the nucellus and in the outer integument. The maize B_sister_ gene *ZMM17* is initially expressed broadly in all organ primordia of the female spikelet, but at later developmental stages expression is restricted to the ovule and the developing silk [6]. Three B_sister_ genes, *OsMADS29*, *OsMADS30,* and *OsMADS31,* were identified in the rice genome [14]. Both RT-PCR and microarray analyses showed that transcription of *OsMADS29* is restricted to developing seeds, *OsMADS30* is expressed throughout all organs of the rice plant, and that expression of *OsMADS31* is below detection limit of the methods being used [14], [15].

Since within angiosperms the monocot rice is only distantly related to the core eudicots *Arabidopsis* and *Petunia*, functional conservation and diversification of B_sister_ genes between monocots and eudicots can be investigated employing these species and along with this provide clues about the ancestral B_sister_ function. Here we investigate *OsMADS29*, because among the three B_sister_ genes of rice it is the only one exclusively and highly expressed during ovule and seed development resembling the expression pattern of B_sister_ genes of other species. We performed detailed expression analyses of *OsMADS29* and explored its functions in rice by a reverse genetics approach employing RNAi. Our results show that *OsMADS29* is mainly expressed in the developing ovule and seed. Knock-down of *OsMADS29* leads to aborted and/or shriveled seeds with deficient accumulation of starch in the endosperm. The severe phenotype of the *OsMADS29* knock-down lines suggests that, it plays an important role during rice seed development and controls at least some aspects non-redundantly with other genes, which is in contrast to typical findings about B_sister_ genes in eudicots. Our results thus corroborate many of the findings recently reported by Yin and Xue (2012) [16]. While these authors, however, focused on transgenic lines with a quite moderate reduction in *OsMADS29* mRNA levels, we report here in more detail the phenotypes of lines with stronger knock-down effects. Moreover, we provide novel insights into the evolution of the B_sister_ gene function and expression in flowering plants and reveal the crucial role of B_sister_ genes in the seed development.

## Results

### Phylogeny of B_sister_ Genes from Monocots

In order to gain insight into the evolutionary relationships between *OsMADS29* and other B_sister_ genes, a comprehensive search for B_sister_ genes of monocots was carried out in GenBank and Phytozome (www.phytozome.net) (Figure S1). A phylogenetic tree was then constructed with the identified genes and representative B_sister_ genes from core eudicots (Figure 1). The topology of the phylogenetic tree shows that B_sister_ genes of core eudicots and monocots constitute two different clades, suggesting that the most recent common ancestor of both taxa contained one B_sister_ gene only. The clade of grass B_sister_ genes is divided into three subclades, termed the *OsMADS29*, *OsMADS30*, and *OsMADS31* subclade. These clades comprise rice B_sister_ genes together with putative orthologs from other grass species such as barely (*Hordeum vulgare*), wheat (*Triticum aestivum*), sorghum (*Sorghum bicolor*), maize (*Zea mays*), *Brachypodium distachyon*, and *Setaria italica* (Figure 1), indicating that the gene duplication events that generated the three clades occurred before grass diversification.

### *OsMADS29* is Specifically Expressed in Ovules and Developing Seeds

To analyze the expression patterns of *OsMADS29* in detail, RT-PCR, quantitative reverse transcription PCR (qRT-PCR), and RNA *in situ* hybridization analyses were performed using diverse tissues at different developmental stages.

**Figure 2:**
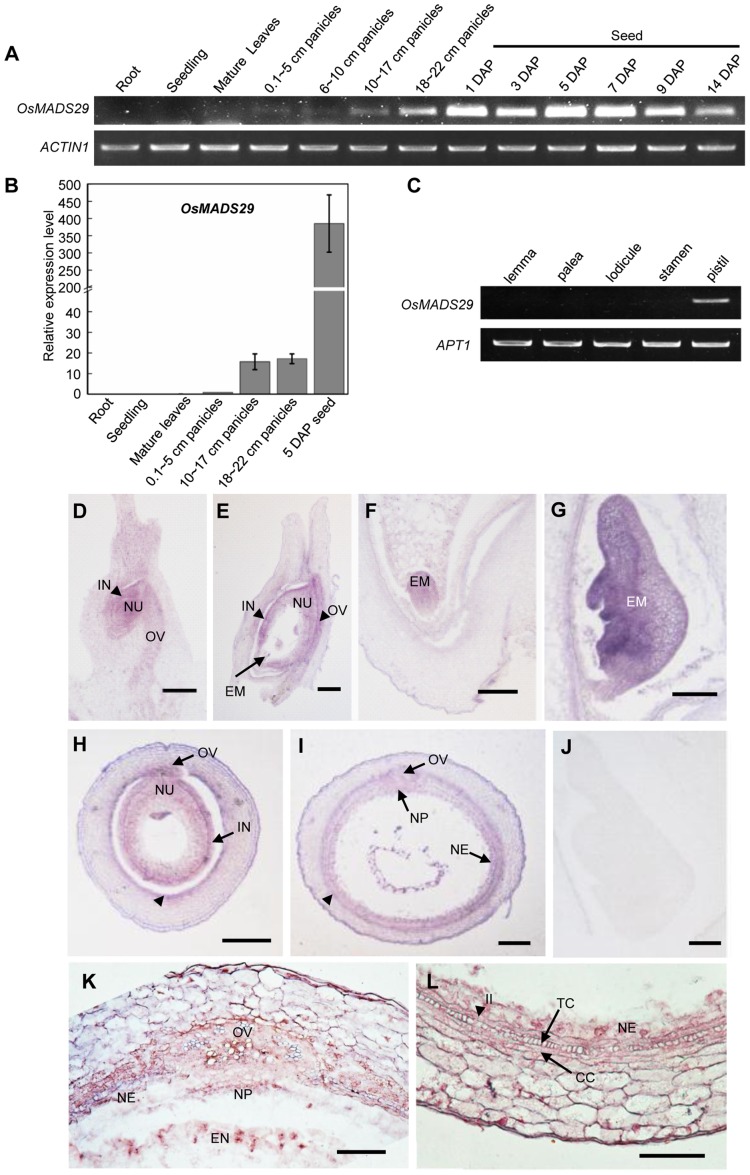
Expression analyses of *OsMADS29*. **(A).** RT-PCR analyses of *OsMADS29* expression at different developmental stages of wild type plants. DAP, days after pollination. *ACTIN1* was used as control. **(B).** Relative expression levels of *OsMADS29* at different developmental stages. Amounts of transcripts are shown as relative values to those of *UBQ*. Error bars show SD (n = 3). **(C).** RT-PCR analyses of *OsMADS29* expression in various floral organs of wild type plants at heading date stage. *APT1* was used as a control [47]. **(D–L).** *In situ* hybridization analyses of *OsMADS29* in ovules and developing seeds. **(D).** Longitudinal section of the ovule. **(E–G).** Longitudinal sections of seed at 1 DAP (E), and embryo of seed at 3 DAP (F) and 5 DAP (G). **(H–I).** Transverse sections of the mid-region of the ovary at a few hours before anthesis (H) and seed at 3 DAP (I). The arrows head in (H) and (I) indicate the inner layer cells of the pericarp. **(J).** Negative control with sense probes of *OsMADS29*. **(K–L).** A magnification of the transverse section of a seed at 7 DAP. (K). The ovular vascular trace in the seed at 7 DAP. (L). Part of pericarp of seed at 7 DAP. CC, cross-cells; EM, embryo; EN, endosperm; IN, integuments; II, inner layer of the inner integument; NE, nucellar epidermis; NP, nucellar projection; NU, nucellus; OV, ovular vascular trace; TC, tube-cells. Bars = 100 µm in (D) to (J), and 50 µm in (K) and (L).

RT-PCR analyses show that the expression of *OsMADS29* initiates at panicles 0.1∼5 cm, then increases gradually with the development of panicles at 6–22 cm. After pollination, the expression level reaches the maximum value at 5 DAP-7 DAP, then decreases from 9 DAP. No expression of *OsMADS29* is detected in the vegetative organs (Figure 2A). Results of qRT-PCR show similar expression profiles of *OsMADS29* to those of the RT-PCR (Figure 2B).

RT-PCR was also performed to examine the expression of *OsMADS29* in different floral organs. Like other known B_sister_ genes, *OsMADS29* is specifically expressed in the pistils (Figure 2C).

*In situ* hybridization was performed to locate the *OsMADS29* expression at the cellular level (Figure 2D–L). Transcripts of *OsMADS29* are localized in the ovule, including integuments (IN) and nucellus (NU) throughout ovule development, from the primordial stage to maturity (Figure 2D and H), as well as in the ovule vasculature. After pollination, *OsMADS29* is expressed evenly throughout the whole seed (Figure 2I and K) and the embryo (EM) (Figure 2E, F and G) except for some cell layers in the outer epidermis of the pericarp. Notably, the gene is expressed in some tissue types which degenerate at later stages of seed development. These tissues include the inner epidermis of the pericarp (Figure 2H and I, indicated by an arrow), the tube-cells (TC) and cross-cells (CC) which differentiate from the inner epidermis at 7 DAP (Figure 2L), the inner and outer integument II (Figure 2D and H), the ovular vascular trace (OV) (Figure 2D, E, H, I and K), the nucellar projection (NP) (Figure 2I, K), and the nucellar epidermis (NE) (Figure 2I, K and L).

### Silencing of *OsMADS29* Results in Seed Abortion and Deficiency in Starch Accumulation in the Endosperm

**Figure 3:**
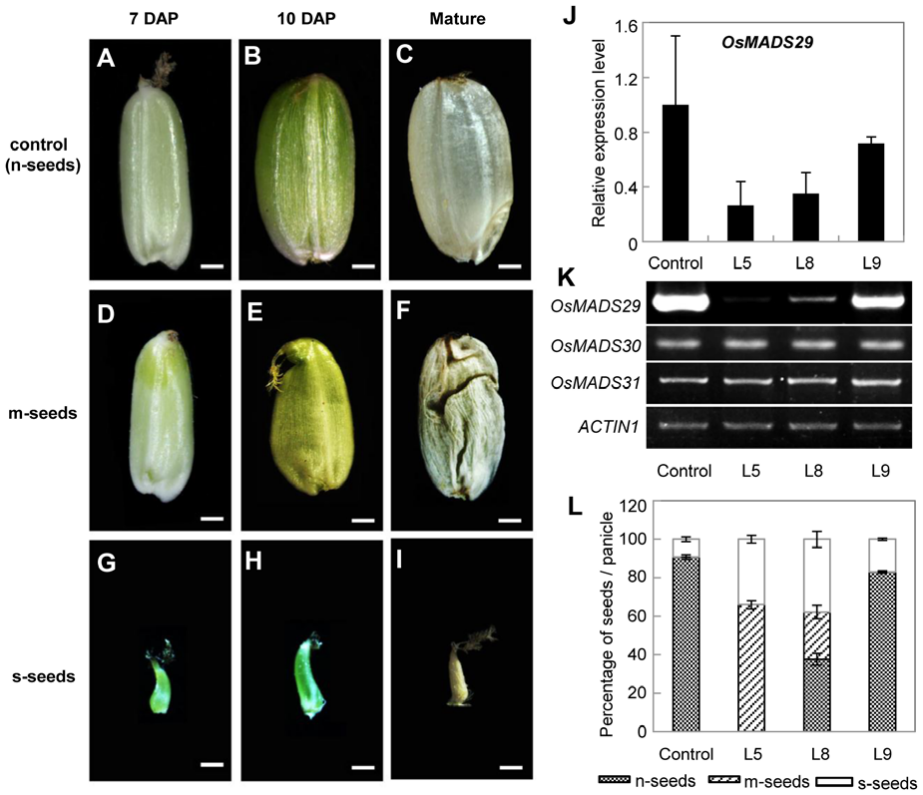
Phenotypes of *OsMADS29* RNAi lines and molecular detections. **(A–I).** Profiles of seeds at 7 DAP, 10 DAP and mature stages, respectively. (A–C), n-seeds of control plants. (D–F), m-seeds with deficient starch accumulation in the endosperm. (G–I), s-seeds that are aborted before 7 DAP. Bars = 1 mm. **(J).** Relative expression levels of *OsMADS29* in control plants and three typical *OsMADS29* RNAi lines (L5, L8 and L9). Total RNA was pooled from stage Ov10 [48]. The *ACTIN1* was used as the internal control. Error bars show SD (n = 3). **(K).** RT-PCR analyses of *OsMADS29* and the other two related B_sister_ genes of rice, *OsMADS30* and *OsMADS31*, in control plants and RNAi plants. Total RNA was pooled from stage Ov10. The *ACTIN1* was used as the internal control. **(L).** Percentage of different kinds of seeds in one panicle in control plants and different *OsMADS29* RNAi lines. The error bar indicates the SE (n = 3).

**Figure 4:**
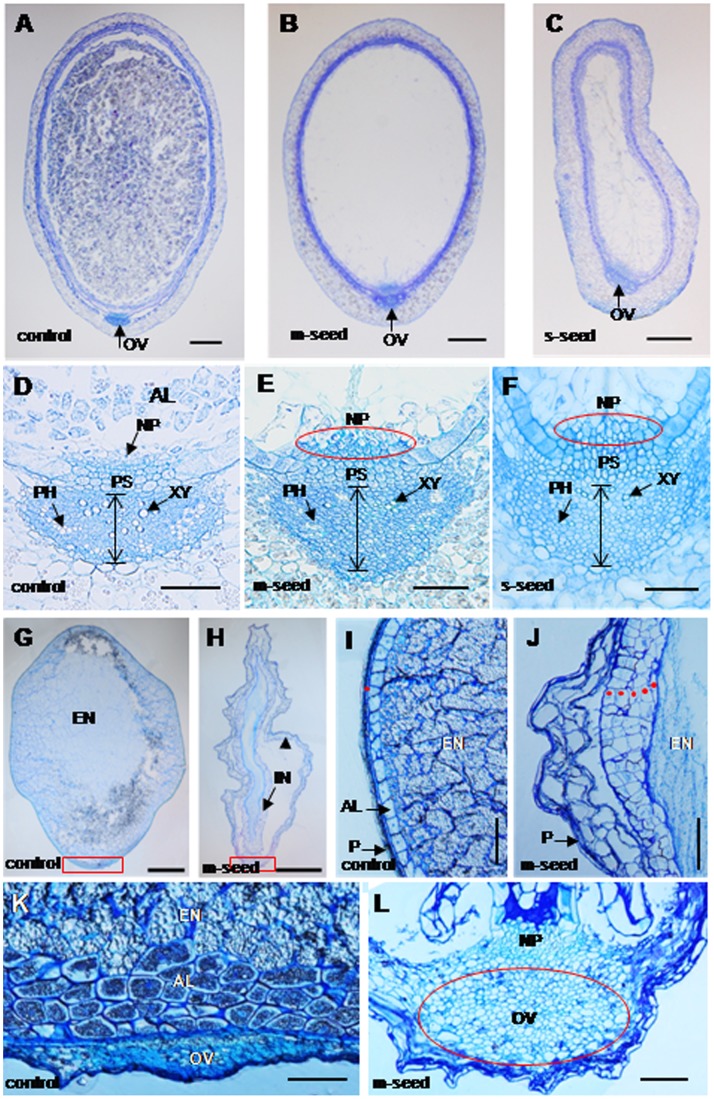
Histological analyses of control and transgenic seeds. **(A).** Starch granules were seen in control seeds at 7 DAP. **(B, C).** m- and s-seeds at 7 DAP without starch granules. **(D–F).** A higher magnification of the OV at 7 DAP in the control seeds (D), m-seeds (E) and s-seeds (F), respectively. The double arrow between the two transverse lines indicates the cell layers of the OV. **(G, H).** Semi-thin transverse sections from the mid-region of mature control seeds (G) and m-seeds (H) at 30 DAP. The red rectangle in (G) and (H) indicates the position of ovular vascular trace. The arrowhead in (H) indicates the remained pericarp. **(I, J).** Higher magnification for part of the pericarp in mature control seeds (I) and m-seeds (J) at 30 DAP. It shows that the pericarp cuticular layer in (I) and persisted pericarp cells in (J). The red dots indicate the cuticular seed coat in control seeds (I) and the cells of integuments and nucellar epidermis in m-seeds are not degenerated (J). **(K, L).** Higher magnification of the OV in control seeds (K) and m-seeds (L), which showed in the red rectangle in (G) and (H). The red circle in (L) indicates the large OV in m-seeds. AL, aleurone; NP, nucellar projecton; OV, ovular vascular trace; P, pericarp; PH, phloem; PS, the pigment strand; XY, xylem; EN, endosperm. Bars = 200 µm in (A–C), 50 µm in (D–F), 500 µm in (G) and (H), 100µm in (I) and (J), and 50 µm in (K) and (L).

To explore the functions of *OsMADS29*, we generated transgenic plants in which *OsMADS29* expression is specifically silenced at different levels by RNAi (Figure 3J and K). Statistical evaluations of data from mature grains shows that there is a usual abortion rate of about 9% in the control plants (Figure 3L). In contrast, of fifteen independent transgenic lines, five lines with strong *OsMADS29* down regulation produced strong phenotypes with 100% abnormal seeds, including, on average, 66% shriveled seeds (mildly affected seeds with deficient starch accumulation in the endosperm, defined as m-seeds) (Figure 3D, E, F, L and Figure 4B) and 34% aborted seeds (severely affected seeds which are fully aborted before 7 DAP, s-seeds) (Figure 3G, H, I, L and Figure 4C). Seven lines with moderate *OsMADS29* down regulation exhibited phenotypes with 63% abnormal seeds, including, on average, 25% m-seeds and 38% s-seeds, and 37% normal grains (n-seeds) (Figure 3L). In the three lines with weak down regulation, most of the grains are n-seeds except for, on average, 17% s-seeds (Figure 3L).

Considering that rice seeds reach their maximum length at about 5–6 DAP [17], 7 DAP were chosen as first time point for investigation of seeds. At 7 DAP, the s-seeds are aborted already (Figure 3G), while the m-seeds are still similar to the control seeds (Figure 3A) except for being slightly smaller in size (Figure 3D). At 10 DAP, unlike n-seeds that show an increasing width, s-seeds did not show any further development (Figure 3H), while the apical part of the m-seeds reveals initial signs of shrinking possibly caused by a failure to accumulate sufficient levels of starch (Figure 3E). One month later, compared with the fully developed n-seeds, the s-seeds did not show much further development compared to 7 DAP (Figure 3I), and the mature m-seeds appear shriveled with wrinkles on their pericarps (Figure 3F).

The agronomic traits of seeds of control and *OsMADS29* RNAi transgenic lines were analyzed except for the s-seeds that had been aborted at 7 DAP (Table 1). In line 5 (L5), in which the *OsMADS29* expression was severely down-regulated, the length of m-seeds is 4.84 mm on average, while control seeds are about 16.7% longer (5.65 mm). The average width of control seeds was 3.15 mm, about 18.9% wider than that of the m-seeds (2.65 mm). The average thickness of m-seeds (1.00 mm) was reduced to about 50% of that of the control (1.98 mm) (P-value <0.01). These differences in length, width, and thickness are probably caused by a deficiency in starch filling. As expected, the 1000-grain-weight is also extremely reduced in m-seeds (2.15 g), with the weight being only about 10% of that of the control seeds (23.48 g) (P-value <0.01).

**Table 1:** Agronomic traits of seeds in control plants and *OsMADS29* RNAi transgenic lines.

	Length (mm)	Width (mm)	Thinkness (mm)	1000-grain-weight (g)
Control (n-seeds)	5.65±0.17	3.15±0.13	1.98±0.06	23.48±0.14
L5 (m-seeds)	4.84±0.23**	2.65±0.17	1.00±0.04**	2.15±0.10**
L8 (m-seeds)	5.61±0.26	2.86±0.18	1.31±0.05**	5.45±0.06**

The 1000-grain-weight of L5 and L8 was measured by using the deficient filled seeds. **P<0.01 when compared with the control seeds.

To correlate the phenotypes with *OsMADS29* transcript abundance, qRT-PCR experiments were performed. As shown in Figure 3J, the transcript level of *OsMADS29* was highly reduced to 26.4% of that of the wild-type in L5 exhibiting a strong phenotype, significantly reduced to 35.3% in L8 with a moderate phenotype, and slightly reduced to 71.7% in L9 showing only a weak phenotype. In contrast, the expression levels of the other two B_sister_ genes in rice, *OsMADS30* and *OsMADS31* remained unchanged in the *OsMADS29* knock-down lines (Figure 3K). These results indicate that the observed phenotypes of the transgenic plants are caused by specific silencing of *OsMADS29.*

Given that the starch content is substantially decreased in mature seeds of transgenic plants (Figure 4B and C), the expression levels of four genes, including *OsAGPS1*, *OsAGPL2*, *OsAGPL3*, and *OsAGPS2a* which encode the rate-limiting-step enzymes in the starch synthesis pathway [18], were examined in developing seeds of control plants and *OsMADS29* RNAi lines at 10 DAP. qRT-PCR results showed that the expression levels of those four genes in transgenic seeds are similar to the ones of the control seeds (Figure S2), indicating that starch synthesis itself is not affected.

### Silencing of *OsMADS29* Affects Vascular Trace Development and the Degeneration of Tissues in Rice Grains

Down regulation of *OsMADS29* does not affect ovule development but causes seeds to abort or shrivel as they are most likely deficient in endosperm development and starch accumulation (Figure S3F, Figure 3A–I, Figure 4B and C). To observe the internal organization of developing seeds, mid-region transverse sections of seeds at 7 DAP were made. While the seeds of control plants are entirely filled with endosperm cells that produce starch granules (Figure 4A), no endosperm cells can be observed in the m- and s-seeds of the transgenic lines at 7 DAP (Figure 4B and C). In control plants, the xylem cells are large, have a circular shape and are centered in the middle of the ovular vascular trace (OV), and the phloem cells are small and arranged in a cluster at the periphery of the OV (Figure 4D). In the s-seeds, the xylem cells are smaller in size and fewer in number than that of the control seeds. Also a lower number of phloem cells can be seen at the margin of the OV (Figure 4F). In m-seeds, the size and number of xylem cells are similar to those of the s-seeds, whereas arrangement style of phloem cells is scattered in contrast to the phloem cell clusters of the control plants (Figure 4E). Moreover, the number of cell layers of the OV in the transverse orientation is higher in the *OsMADS29* RNAi transgenic lines than in the control lines (marked by double arrows brackets in Figure 4D–F), suggesting that adjacent cells in the OV probably fail to integrate into the xylem and phloem. Another tissue type recognized as abnormal in the transgenic lines is the nucellar projection (NP), which is a small zone of persisting nucellar cells attached to the chalaza. The NP cells degenerate in the control seeds at 7 DAP such that of originally 4–5 cell layers only 2–3 layers remain. The respective cells are small and flat, and constitute a tissue required for nutrient transfer to the endosperm and embryo (Figure 4D; Figure S4). In contrast, the NP cells of m- and s-seeds at 7 DAP remain large and circular, without any obvious indication of cell degeneration (Figure 4E and F).

**Figure 5:**
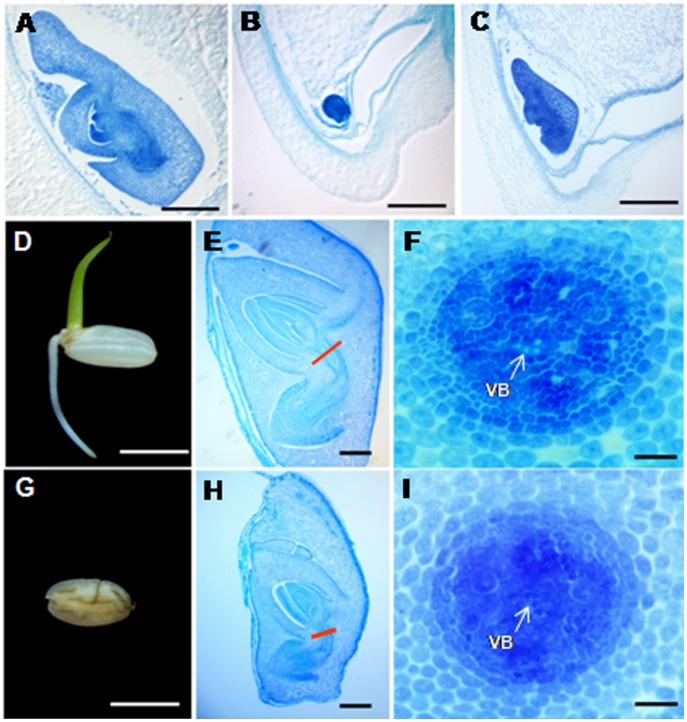
Histological structure of embryo and germination of mature seeds. **(A).** Longitudinal sections of control embryo at 7DAP. **(B, C)** Longitudinal sections of embryo of s-seeds (B) and m-seeds (C) at 7 DAP, respectively. **(D).** The normally germinated seed of control plant. **(E).** Longitudinal sections of the mature embryo in control seed. The red line referred to the position of vascular bundle. **(F).** Magnifying observation of cross sections from the red line marked parts in (E). The arrow showed the clear vascular bundle. **(G).** The m-seeds failed to germinate. **(H).** Longitudinal sections of the mature embryo in m-seeds. **(I).** Magnifying observation of cross sections from the red line marked parts in (H). No obvious vascular bundles were observed at the arrow referred place. VB, vascular bundles. Bars = 20 µm in (A–C), 5 mm in (D) and (G), 0.2 mm in (E) and (H), 20 µm in (F) and (I).

In mature grains, the structure of the embryo was not affected in the m-seeds, except for the fact that the embryos were considerably smaller than in control seeds (Figure 5E and H). Notably in the n-seeds at 30 DAP, the maternal tissues (pericarp, ovular vascular trace, integuments, and nucellar epidermis) were almost degenerated to form the cuticula (Figure 4G, I and K) along with the maturity of filial tissue (endosperm and embryo). In contrast, all the maternal tissues remained in the mature seeds in the *OsMADS29* RNAi transgenic lines at this stage (Figure 4H, J and L).

Longitudinal histological sections of seeds at 7 DAP showed that development of the embryo in transgenic seeds severely lagged behind that of the wild-type. Compared to the completed embryo morphology in control seeds at 7 DAP (Figure 5A), embryos of s-seeds at 7 DAP appear to cease development at a stage corresponding to 3 DAP of wild-type seeds, and remain in this stage (Figure 5B and Figure S3C); embryos of m-seeds at 7 DAP grew retarded to a stage corresponding to almost 5 DAP of control seeds (Figure 5C and Figure S3D). In mature grains, the embryo of m-seeds is considerably smaller than the one in control seeds (Figure 5E and H). Considering that the mature embryo of m-seeds failed to germinate even on the 1/2 MS culture medium (Figure 5G), mid-region transverse sections of embryos were carried out to thoroughly observe the structure of the vascular system, which also developed abnormally in m- and s-seeds at 7 DAP. While vascular bundles are obvious between the shoot apical meristem and radicle of control embryos, no vascular bundles were observed in the embryos of m-seeds (Figure 5F and I).

**Figure 6:**
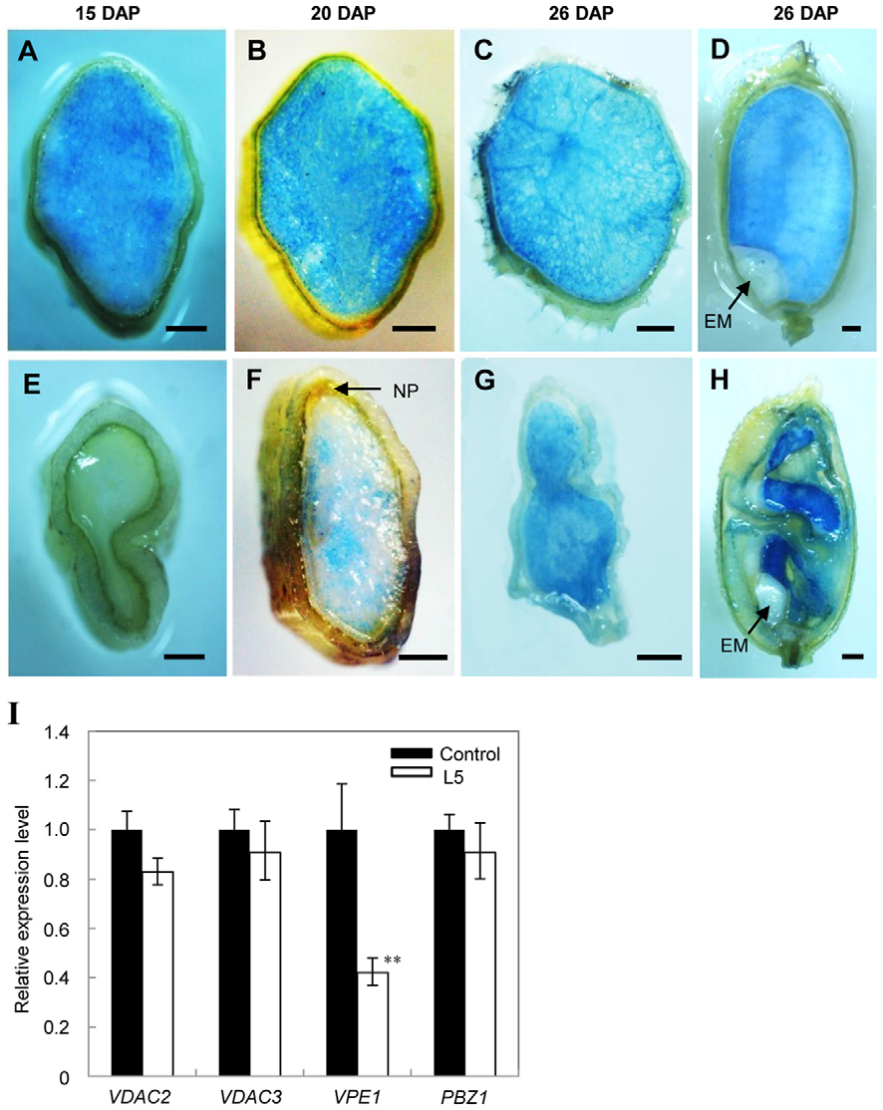
Detections of tissue degeneration. **(A–D).** Evan’s Blue staining of control seeds. (A-C) Transverse sections at 15 DAP, 20 DAP, and 26 DAP, respectively. (D) The longitudinal section at 26 DAP. The blue tissues indicate dead endosperm cells. **(E–H).** Evan’s Blue staining of m-seeds. (E–G) Transverse sections at 15 DAP, 20 DAP, and 26 DAP respectively. H Longitudinal sections at 26 DAP. Bar = 500 µm in (A) to (H). EM, embryo. **(I).** Quantitative real-time PCR analyses of PCD-related genes in 7 DAP seeds of control and transgenic plants. *ACTIN1* was used as an internal control. Error bar indicated the SD (n = 3). ** indicates an extremely significant difference (P<0.01) between control and transgenic lines.

Previous studies have shown that the differentiation of the tracheary elements as well as the degeneration of nucellus and pericarp cells are also brought about by PCD [19]–[21]. To analyze the relationship between *OsMADS29* and cell degeneration caused by PCD during seed development, an Evan’s blue staining experiment, which can dye the dead cells to be blue, was performed. Results showed that cell death of the NP and endosperm tissues in the m-seeds obviously occurs later than that of control seeds during the stage from 15 DAP to 26 DAP (Figure 6A–H). In addition, qRT-PCR expression analyses were carried out with three classes of genes known to positively regulate PCD through three different pathways, including *VPE1-4* [22]–[24], *VDAC1-3* [25], [26], and *PBZ1* [27], [28]. Our results show that *OsVPE1* (Os04g45470) is the only gene which is significantly down-regulated in *OsMADS29* knock-down transgenic seeds (Figure 6I). A putative CArG-box with the consensus sequence C(A/T)_8_G was found at position −1827 in the upstream region of *OsVPE1* (Figure S5). As MADS-domain proteins have been shown to bind to CArG-boxes of their target genes to regulate expression [29], this may indicate direct regulation of *OsVPE1* by *OsMADS29*.

## Discussion

### *OsMADS29* is a Canonical B_sister_ Gene in Rice

As revealed by phylogenetic analyses previously, B_sister_ genes are the sister clade of the *DEF*/*GLO*-like (or *AP3*/*PI*-like) genes comprising class B floral organ identity genes [6]. Our phylogeny suggest that the two gene duplications that gave rise to the three B_sister_ genes *OsMADS29*, *OsMADS30* and *OsMADS31* in rice occurred after the divergence of the lineages that led to extant eudicots and monocots about 150 MYA [30], but predated the diversification of grasses from a common ancestor about 55–70 MYA [31]–[33]. In our phylogeny, the branch lengths are relatively short for all genes belonging to the *OsMADS29* clade as compared to the branch lengths for the genes belonging to the other two monocot B_sister_ clades (Figure 1). This suggests that purifying selection is acting on the genes in the *OsMADS29* clade. Furthermore, the expression pattern of the genes in the *OsMADS29* subclade seems to be conserved, as the expression of *OsMADS29* in the developing ovule including integuments and nucellus is similar to that of the previously described expression patterns of *WBsis* and *ZMM17* (Figure1) [6], [7]. Taken together, this provides evidence for the functional importance of the genes in the *OsMADS29* clade. Protein sequence alignments of the C-terminal domains show that OsMADS29 and OsMADS31 are typical B_sister_ proteins with respect to their protein sequence as they both share a conserved sub-terminal ‘PI Motif-Derived sequence’ and a terminal ‘Paleo AP3 Motif’ with other B_sister_ proteins. In contrast, OsMADS30 does not include these motifs characteristic for B_sister_ proteins (Figure S6). Expression analyses reveal that *OsMADS31b* (the alternative splice isoform of *OsMADS31* reported by Lee *et al*. (2003) [15]) is broadly expressed during all developmental stages of rice, whereas *OsMADS30* expression is hardly detectable at all (Figure S7), which are unusual expression patterns for B_sister_ genes. Only *OsMADS29*, which is specifically expressed in mature florets, especially in pistils and in developing seeds, exhibits an expression pattern typical for B_sister_ genes (Figure 2A–C). After pollination, *OsMADS29* is highly expressed in developing seeds (Figure 2D–L). Expression during seed development has been reported for *ABS*, *GOA* and *FBP24* as well [8]–[10]. These results suggest functional conservation of B_sister_ genes between eudicots and monocots in ovule and seed development. Furthermore, our study shows that *OsMADS29* is probably the canonical representative of these functions in rice.

### Conserved Functions of B_sister_ Genes in Ovule and Seed Development

Given that all B_sister_ genes investigated so far, including *OsMADS29*, are expressed during early ovule development, it was hypothesized that members of the B_sister_ subfamily play important roles in female reproductive organ (ovule) development [34]. In our study, down-regulation of *OsMADS29* does not affect the structure of the ovule (Figure S3A and F). Accordingly, either *OsMADS29* is not required for ovule specification in rice or its function in ovule development is obscured by redundancy with another gene, e.g. with *OsMADS21*, which is a class D gene. The expression of *OsMADS21* overlaps with *OsMADS29* and single mutants of *osmads21* do not have a mutant phenotype [35]. Redundancy between B_sister_ and D class genes would resemble recent findings for *ABS/TT16* and *STK* in *Arabidopsis* [13]. Redundancy with the other two B_sister_ genes *OsMADS30* and *OsMADS31* may also obscure the spectrum of functional significance of *OsMADS29* during ovule development. Recently, some microarray data about ovule development can be used from the public database, which can give us some new clues for the ovule development of *OsMADS29* in our future work [36].

In contrast to ovule development, the effect on seed development is stronger for *OsMADS29* than for the *Arabidopsis* B_sister_ genes. While the phenotypes of the single mutants *abs* (*tt16*) and *goa* of *Arabidopsis* are mild, given that they still produce seeds which germinate properly [9], [11], [12], silencing of *OsMADS29* in rice leads to a severe phenotype with sterile seeds which are aborted or shriveled. The mild phenotype of the *abs* single mutant may again be explained by redundancy of *ABS* (*TT16*) and *STK* in *Arabidopsis* [13]. In rice, this redundancy between B_sister_ and class D genes in seed development may have been lost or not gained in evolution, leading to a severe phenotype in *OsMADS29* knock down lines. Compared to control seeds which are full of starch granules, cross-sections of abnormal seeds at 7 DAP show no visible starch granules. Interestingly, starch accumulation is also deregulated in the *abs*/*stk* double mutant. However, in this case, an excess of starch was observed in the embryo sac and the developing seed [13]. Although some shriveled seeds of the *OsMADS29* knock down lines have embryos with normal shape, they have no vascular bundles and fail to germinate, possibly because of the lack of nutrition transport (Figure 5D–I).

The mutant phenotypes of B_sister_ genes from eudicots and rice differ during ovule and seed development. This may indicate that the function of *OsMADS29* has diverged from that of B_sister_ genes in eudicots during the evolution of monocots. However, the differences in the mutant phenotypes can also, at least partially, be explained by redundancy to other genes. Hence, it is also possible that the function of *OsMADS29* resembles the ancestral function of B_sister_ genes while this ancestral function is masked in eudicots due to other genes with redundant functions.

### *OsMADS29* is Required for Vascular Trace Development and Cell Degeneration in Rice Seeds

Previous studies have shown that there is a symplastic continuity, including the ovular vascular trace (OV), pigment strand (PS) and nucellar projection (NP), which is the nutrient source for the filial tissues (endosperm and embryo), between the rice maternal tissues and the filial tissues [37]–[39]. Therefore the path among the OV, PS and NP plays an important role in grain filling and seed development. In control plants, the nucellus was absorbed by the filial tissues during early seed development (Figure 4D). In contrast, the structures of the nucellus, including NP and NE are still visible in the transgenic grains at 7 DAP and in mature seeds (Figure 4E, F, H, J and L). This indicates that the process of degeneration of their cells is partially suppressed. Similar results concerning the defect structure of NE and NP have recently been reported by Yin and Xue (2012) when this manuscript was in preparation [16]. However, we also observed defects in other tissues, such as the OV, which may lead towards a more comprehensive understanding of the function of *OsMADS29.* In the transgenic abnormal seeds at 7 DAP, the shapes of the OV are irregular and the cell numbers of xylem and phloem are less than those in the control plants (Figure 4E, F), indicating that transport paths for nutrients are strongly affected. Xylem and phloem are differentiated from the cambium cells, revealing that their formation is a process involving PCD [21]. Hence, fewer cells in the xylem and phloem of transgenic abnormal seeds at 7 DAP imply that cell degeneration of the tracheary tissue (including xylem and phloem) is suppressed to a great extent. This further indicates that the defect tracheary system may be another important cause, besides the failed degradation of NE and NP, for the deficient starch accumulation, as no tracheary system is observed in the m-seeds at mature stage. Therefore, the loss of nutrient transport may be the possible direct reason for the small embryo and the germination failure. This result also shows that *OsMADS29* affects not only the cell degeneration of maternal tissues but also that of filial tissues. Furthermore, the pericarp cells in the *OsMADS29* transgenic m-seeds remain in their original state without any sign of degeneration (Figure 3B and E). However, the pericarp of the wild type mature seed is degenerated in a PCD dependent process to become the cuticula which protects the filial tissue (Figure 4I) [19]. Collectively, these results show that the degeneration of cells is localized in those tissues of seeds where gene expression of *OsMADS29* is found.

As expected, cell degeneration was indeed shown to be suppressed during seed development of transgenic plants. Evan's blue staining indicates that cell death of NP and endosperm tissue in transgenic seeds is obviously delayed as compared to that of control seeds (Figure 6A–H), suggesting that the process of PCD is delayed and suppressed to some extent. Since molecular markers for cell death in seeds are not available at present, the expression of three kinds of genes, *VPEs*, *VADC*, and *PBZ1*, known to be involved in PCD through different pathways, was investigated. Previous studies showed that the *VPEs* are involved in vacuole mediated cell death in both defense and development, regulating PCD in *Arabidopsis*, rice, and barley [22]–[24]. Only four *VPE* genes were found in rice, among which *OsVPE1* and *OsVPE4* are seed-type genes, whereas *OsVPE2* and *OsVPE3* are vegetative-type genes [22]. In this study, only the transcript amount of *OsVPE1* was found to be substantially reduced in abnormal transgenic seeds (Figure 6I). In contrast, the expression levels of the *VDAC* genes, which are elements of the mitochondrial death machinery [25], [26], and *OsPBZ1*, which is involved in PCD induced by abiotic stresses [27], [28], were the same in abnormal transgenic as in control seeds (Figure 6I). A Cys protease involved in regulating PCD of maternal tissues has been identified as a direct target gene of *OsMADS29* by Yin and Xue (2012), but the pathway of PCD regulated by *OsMADS29* has remained unknown [16]. Our molecular analyses together with the histological structures of the abnormal seeds in our transgenic plants suggest that PCD of the tracheary system and pericarp in rice caryopses regulated by *OsMADS29* occurs through a pathway mediated by vacuolar processing. The expression levels of genes encoding the rate-limiting-step enzymes of the starch synthesis pathway, *OsAGPS*1, *OsAGPS2a*, *OsAGPL2*, and *OsAGPL3*, are not affected (Figure S2). Taken together, our results reveal that the failure of the degeneration process in maternal tissues and thus insufficient development of xylem and phloem leading to an undersupply of filial tissue, rather than the starch synthesis process itself, is the original cause for the abnormal development of transgenic seeds. Interestingly, as mentioned above, also the eudicot B_sister_ genes *ABS* and *FBP24* are functioning in the maternal tissue that supplies the filial tissue with nutrients [8], [9], [12], [13]. Hence, developmental control of the tissue responsible for nutrient supply to the embryo may be one of the ancestral functions of B_sister_ genes.

Our molecular analyses and structural observations provide evidence for the function of *OsMADS29* in cell degeneration during seed development and may enable a better understanding of the process of PCD during seed development. The severe phenotype of *OsMADS29* knockdown lines indicates that this B_sister_ gene at least in part functions non-redundantly to other genes during seed development which is in contrast to the B_sister_ genes of eudicots which have been studied so far. Furthermore, the expression pattern of *OsMADS29* is similar to that of the gymnosperm B_sister_ gene *GGM13* and resembles, more or less, a combination of the expression patterns known from eudicot B_sister_ genes. Hence, *OsMADS29* may have largely retained the ancestral expression pattern and its functions as revealed here may represent the ancestral function of B_sister_ genes. On the other hand, different functions and regulatory mechanisms of eudicot B_sister_ genes in cell expansion, pigment accumulation or integument specification suggest that B_sister_ genes may have undergone sub- and neo-functionalization in various species during the evolution of flowering plants to control different aspects of seed development.

## Methods

### Plant Materials

The rice cultivar ‘Zhonghua 11′ (*Oryza sativa* L. ssp. *japonica*) was planted in local paddy-fields of the Institute of Botany, the Chinese Academy of Sciences.

### Knockdown Vector Construction of *OsMADS29* and Rice Transformation

The *OsMADS29* full length cDNA clone (Accession NO. AK109522) was obtained from the Japan NIAS KOME stock center (http://cdna01.dna.affrc.go.jp/cDNA/). To generate the *OsMADS29* knock-down vector, a 488 bp long specific region (nucleotide positions 474 - 962 counted from ATG) was amplified, using the PCR primer pairs 29RNAiF (*Bam* HI and *Spe* I) and 29RNAiR (*Nco* I and *Hpa* I). The intron and nos terminator cassette of pJawohl3-RNAi (GenBank Accession No. AF404854) was transferred with *Bam* HI/*Not* I site to the pBluescript SK (Stratagene, La Jolla, CA, USA), termed pBJWl3 as an intermediate vector. The RNAi-fragment of *OsMADS29* was cloned into two sides of the intron region of the pBJWl3 vector in sense (*Bam* HI/*Nco* I) and antisense (*Spe* I/*Hpa* I) orientation, respectively. Finally, the dsRNAi cassette containing two oppositely orientated coding sequences and the nos terminator was mobilized with restriction enzymes *Bam* HI and *Sac* I, and was introduced into the vector pCAMBIA1301-Ubi, in which the maize (*Zea mays*) *Ubiquitin* promoter was inserted using the *Hind* III and *Bam* HI sites, resulting in the final plasmid pUOsM29I. The construct was verified by sequencing and restriction mapping. Rice transformation was performed according to the previous methods [40].

### *In situ* Hybridization

Fresh wild-type flowers and seeds (1–7 DAP) were fixed immediately and embedded in paraffin (Sigma). 8 µm-thick sections were hybridized with the *OsMADS29* specific sequences (nucleotides 695–962 counted from the start codon ATG) according to the method described by Cui *et al*., (2010) [40].

### RT-PCR and Quantitative Real-time RT-PCR Analyses

Total RNA was isolated from different rice tissues using TRIzol reagent (Invitrogen, Carlsbad, USA) according to the manufacturer’s instructions. Reverse transcription was performed using Superscript-III Reverse Transcriptase (Invitrogen, Carlsbad, USA). The diluted cDNA samples were used as templates for RT-PCR and real-time PCR. The internal control genes for RT-PCR were *ACTIN1* and *APT1* (*Adenine Phosphoribosyltransferase1*). Real-time PCR were performed using SYBR Premix Ex Taq (Takara, Dalian, China) on a Rotor-Gene 3000 (Corbett Research, QIAGEN, Hilden, Germany) detection system and software according to the manufacturer’s instructions. The *ACTIN1* and *UBQ* were used as an internal control. Gene-specific primers are shown in Table S1.

### Histochemical Analyses

Samples were embedded in paraffin (Sigma-Aldrich, St. Louis, USA) for histological sections (8-µm thickness) and in LR white resin (Sigma-Aldrich, St. Louis, USA) for semi-thin slices (1-µm thickness), respectively. Slices were stained with 0.1% toluidine blue and observed with a light microscope.

### Evans Blue Staining

Fresh caryopses at different days after pollination were cut into longitudinal and cross-sections with a sharp double-edged blade. The sections were stained with Evans Blue solution (0.1% in H_2_O) for 2 min and washed with water for 60 min, then photographed using a Leica microscope (Leica DM4500B).

### Phylogenetic Analyses

B_sister_ genes from monocotyledonous plants were searched using the blastn algorithm (Figure S1). The amino acid sequences of the three B_sister_ proteins from *Oryza sativa*, OsMADS29, OsMADS30 and OsMADS31, were used as query sequences. The databases “Nucleotide collection (nr/nt)” and “non-human, non-mouse ESTs (est_others)” were searched. Furthermore, the whole genome sequences of *Setaria italica*, *Brachypodium distachyon*, *Sorghum bicolor* and *Panicum virgatum* were searched at phytozome (www.phytozome.net). The BLAST results were combined with known B_sister_ genes and representative MIKC-type MADS-box genes of other clades in a neighbor joining tree constructed using PAUP* version 4.0b10 [41] to identify B_sister_ genes and to remove redundancies. Amino acid sequences of known B_sister_ proteins and the newly identified B_sister_ proteins were aligned with ProbCons [42]. The ProbCons alignment was reverse translated into a nucleotide alignment using RevTrans1.4 [43]. The best fitting nucleotide substitution model to this alignment was determined using PAUP* version 4.0b10 [41] and Modeltest3.7 [44] where positions 1 to 24 and 508 to the end of the alignment were excluded. With the best-fitting model of nucleotide substitution (GTR+I+G) [45], a MrBayes v3.1.2 [46] phylogeny was determined, excluding the same positions as for the Modeltest, using AP3 of *Arabidopsis thaliana* as outgroup, generating 3,000,000 trees, sampling every 100th generation and discarding 7,500 of the sampled trees. The final phylogeny is a completely resolved consensus tree with Bayesian posterior probabilities.

## Supporting Information

Figure S1 Accession numbers of genes used in the phylogeny trees.
(TIF)

Figure S2 Expression detections for the selected genes related to the starch synthesis. Quantitative real-time PCR analyses of ADP-glucose pyrophosphorylase genes in young seeds (10 DAP) of control and RNAi transgenic plants. *ACTIN1* was used as an internal control. Error bars indicate the SD (n = 3).
(TIF)

Figure S3 Longitudinal sections of ovule and embryo of control and RNAi transgenic plants. (**A**)**.** Longitudinal sections of mature ovule (OV10) in control plant. (**B–E**)**.** Longitudinal sections of embryo of developing seeds in control plants at 1, 3, 5, and 7 DAP, respectively. (**F**)**.** Longitudinal sections of mature ovule (OV10) in RNAi transgenic plant. Bar = 20 µm.
(TIF)

Figure S4 Transverse sections in the mid-region of control seeds at 0–7 DAP. (**A–C**)**.** Transverse sections of control seeds at stages 0, 1, 2 DAP. (**D–F**)**.** Magnification of OV and NU at stages 0, 1, 2 DAP, respectively. (**G–H**)**.** Transverse sections of control seeds at stages 3, 5, 7 DAP, respectively. (**J–I**)**.** Magnification of OV and NP at stages 3, 5, 7 DAP, respectively. NP, nucellar projection; NU, nucellus; OV, ovular vascular trace. Bar = 50 µm in (A–C), 25 µm in (D–F), 100 µm in (G–H), 200 µm in I, and 50 µm in (J–L), respectively.
(TIF)

Figure S5 The promoter of *OsVPE1* upstream about 3000 bp. A putative CArG-box at position -1827 in the upstream region of *OsVPE1.*
(TIF)

Figure S6 C-terminal sequence alignment of B_sister_ Proteins.
(TIF)

Figure S7 RT-PCR analyses of *OsMADS30* and *OsMADS31*. (**A**). RT-PCR analyses of *OsMADS30* and *OsMADS31* at different development stages. DAP, days after pollination. *ACTIN1* was used as control. (**B**). RT-PCR analyses of *OsMADS30* and *OsMADS31* expression in various floral organs of wild type plants at heading date stage. *APT1* was used as a control.
(TIF)

Table S1 Gene specific primers used in this study.
(DOCX)
