# Supplementary figures and images for: Live and Let Die - The Bsister MADS-Box Gene OsMADS29 Controls the Degeneration of Cells in Maternal Tissues during Seed Development of Rice (Oryza sativa)
Source: PLoS One. 2012 Dec 12;7(12):e51435. doi: 10.1371/journal.pone.0051435 (PMC3520895; doi:10.1371/journal.pone.0051435)

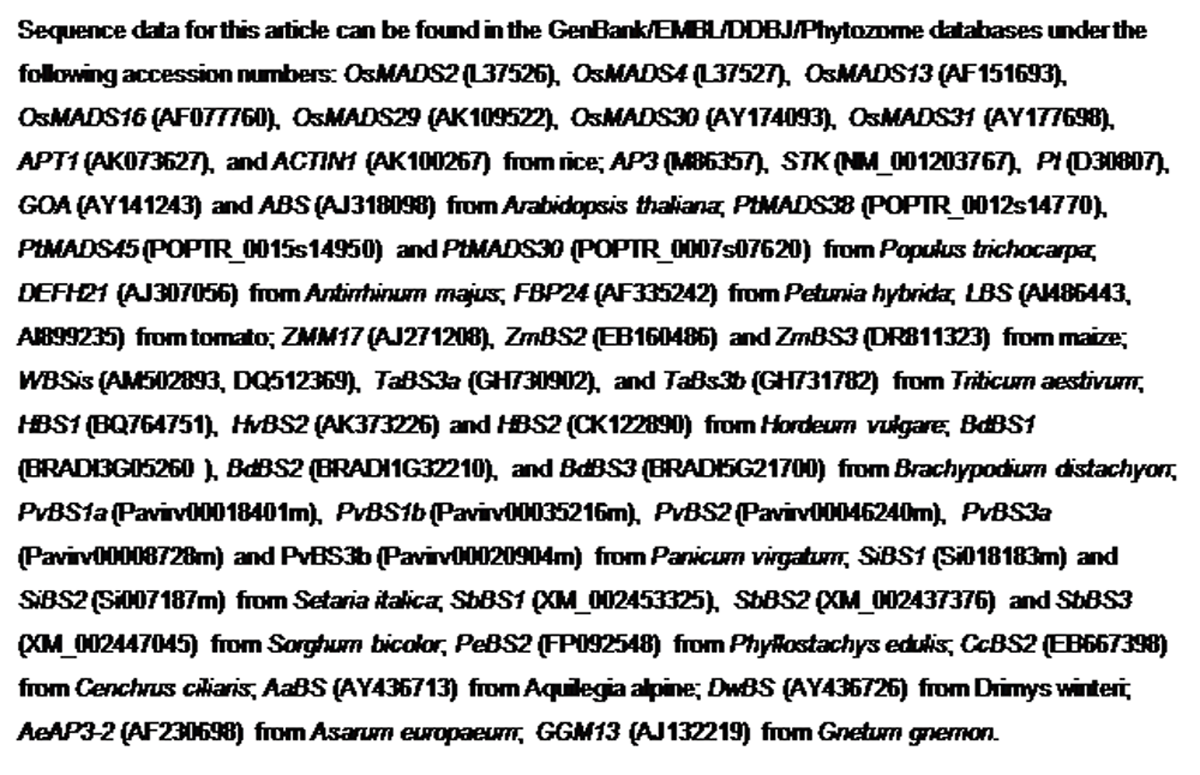

Supplement: Figure S1 — Accession numbers of genes used in the phylogeny trees. (TIF) [file pone.0051435.s001.tif]

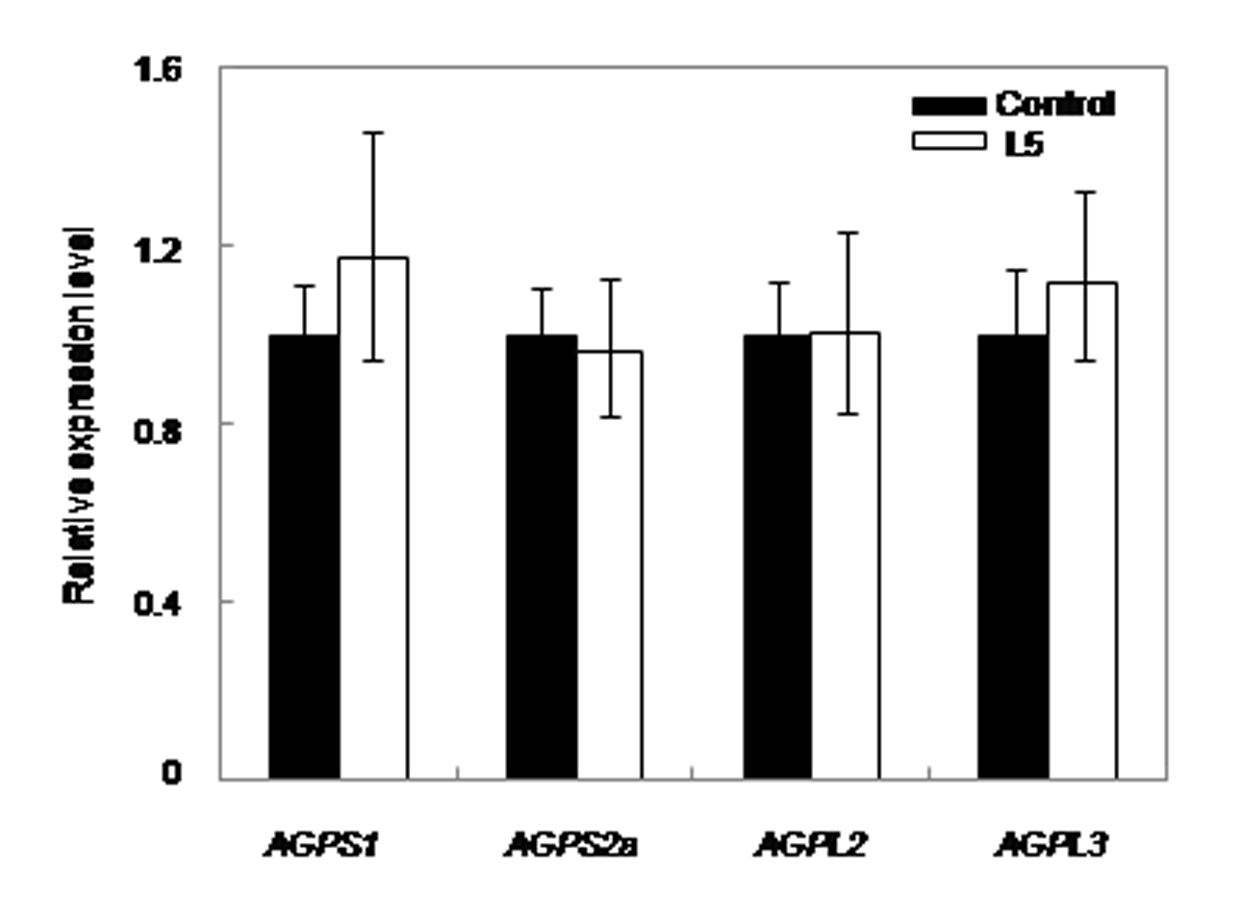

Supplement: Figure S2 — Expression detections for the selected genes related to the starch synthesis. Quantitative real-time PCR analyses of ADP-glucose pyrophosphorylase genes in young seeds (10 DAP) of control and RNAi transgenic plants. ACTIN1 was used as an internal control. Error bars indicate the SD (n = 3). (TIF) [file pone.0051435.s002.tif]

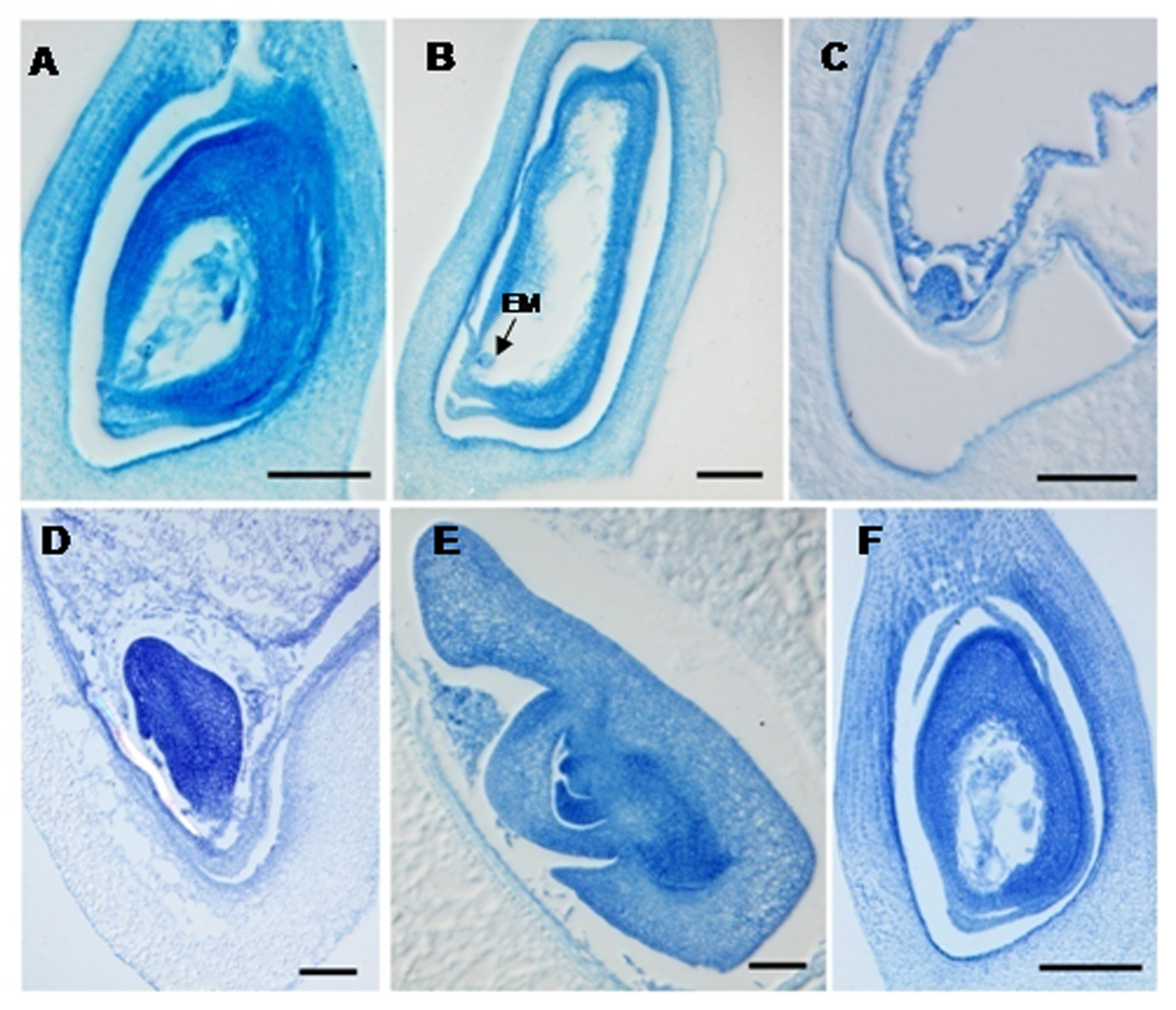

Supplement: Figure S3 — Longitudinal sections of ovule and embryo of control and RNAi transgenic plants. (A). Longitudinal sections of mature ovule (OV10) in control plant. (B–E). Longitudinal sections of embryo of developing seeds in control plants at 1, 3, 5, and 7 DAP, respectively. (F). Longitudinal sections of mature ovule (OV10) in RNAi transgenic plant. Bar = 20 µm. (TIF) [file pone.0051435.s003.tif]

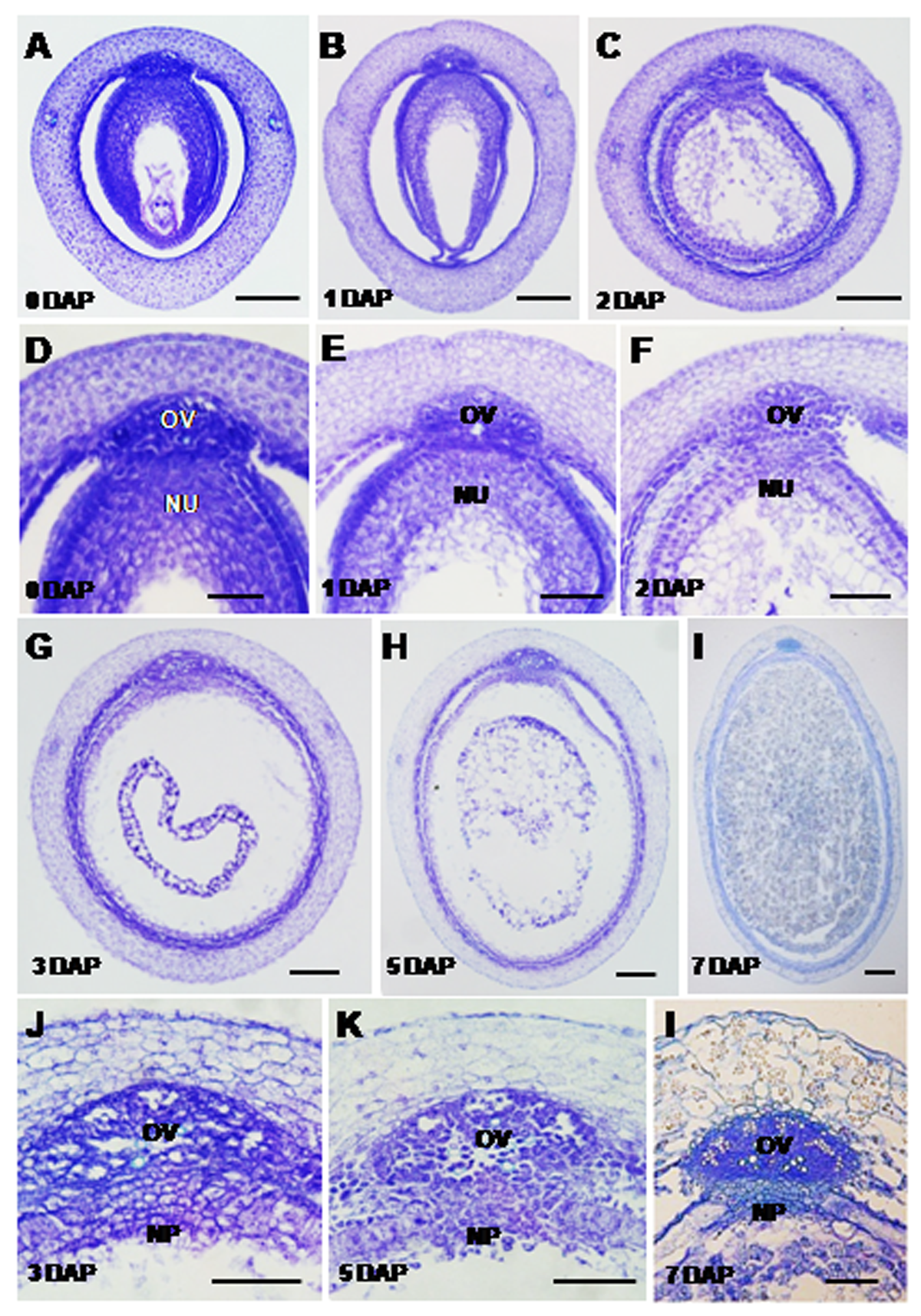

Supplement: Figure S4 — Transverse sections in the mid-region of control seeds at 0–7 DAP. (A–C). Transverse sections of control seeds at stages 0, 1, 2 DAP. (D–F). Magnification of OV and NU at stages 0, 1, 2 DAP, respectively. (G–H). Transverse sections of control seeds at stages 3, 5, 7 DAP, respectively. (J–I). Magnification of OV and NP at stages 3, 5, 7 DAP, respectively. NP, nucellar projection; NU, nucellus; OV, ovular vascular trace. Bar = 50 µm in (A–C), 25 µm in (D–F), 100 µm in (G–H), 200 µm in I, and 50 µm in (J–L), respectively. (TIF) [file pone.0051435.s004.tif]

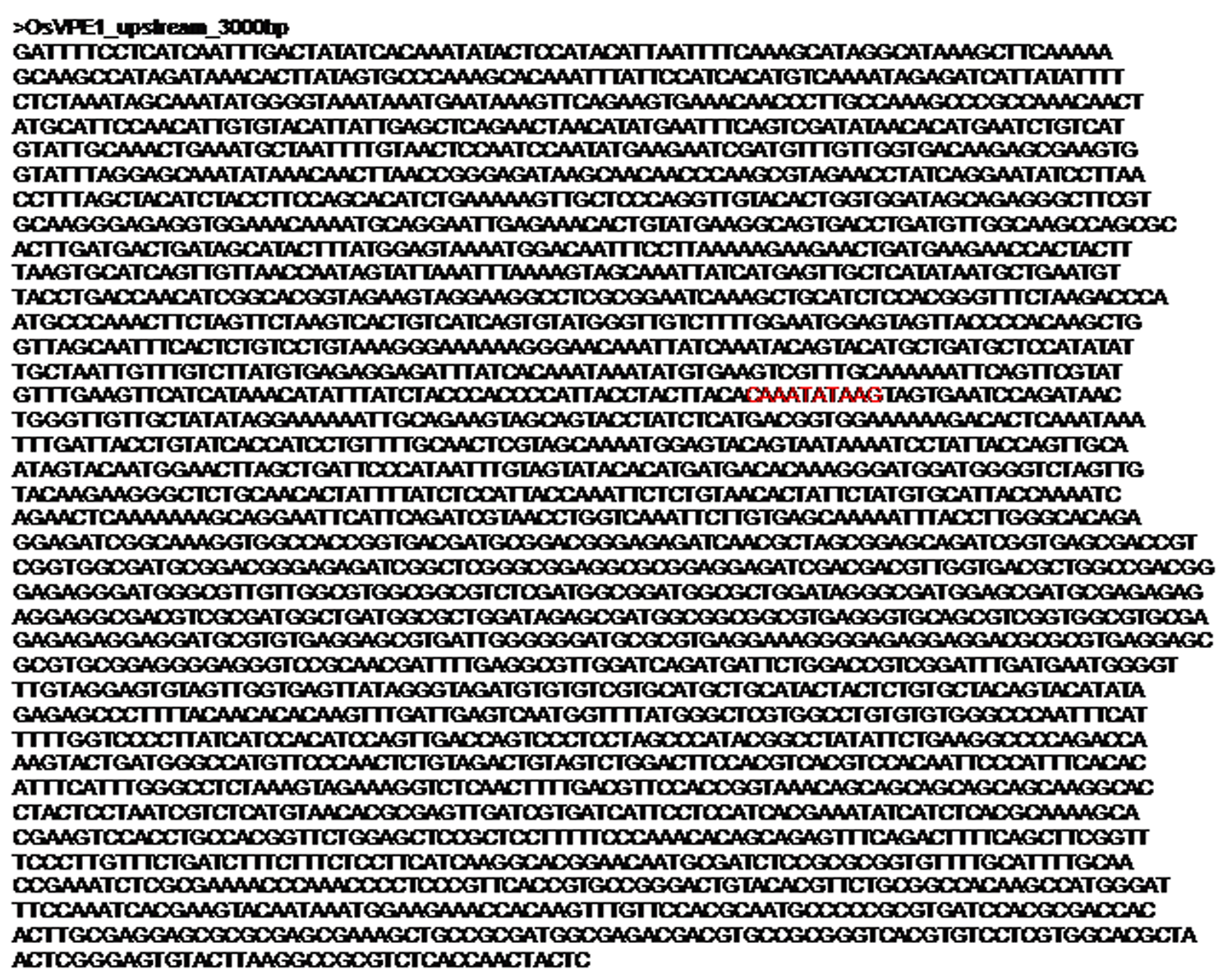

Supplement: Figure S5 — The promoter of OsVPE1 upstream about 3000 bp. A putative CArG-box at position -1827 in the upstream region of OsVPE1. (TIF) [file pone.0051435.s005.tif]

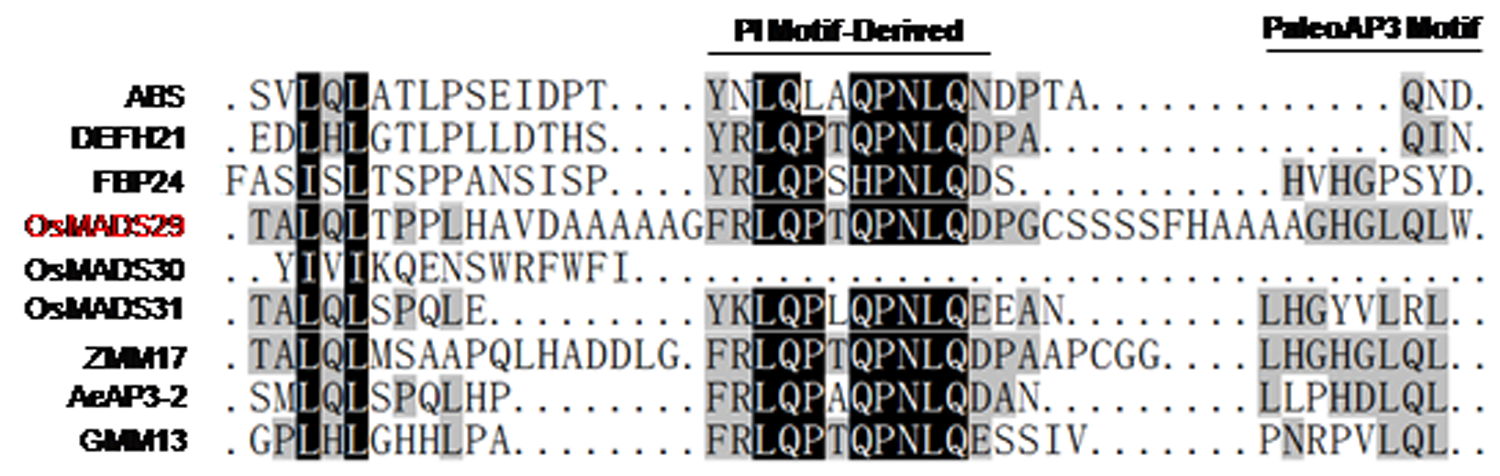

Supplement: Figure S6 — C-terminal sequence alignment of Bsister Proteins. (TIF) [file pone.0051435.s006.tif]

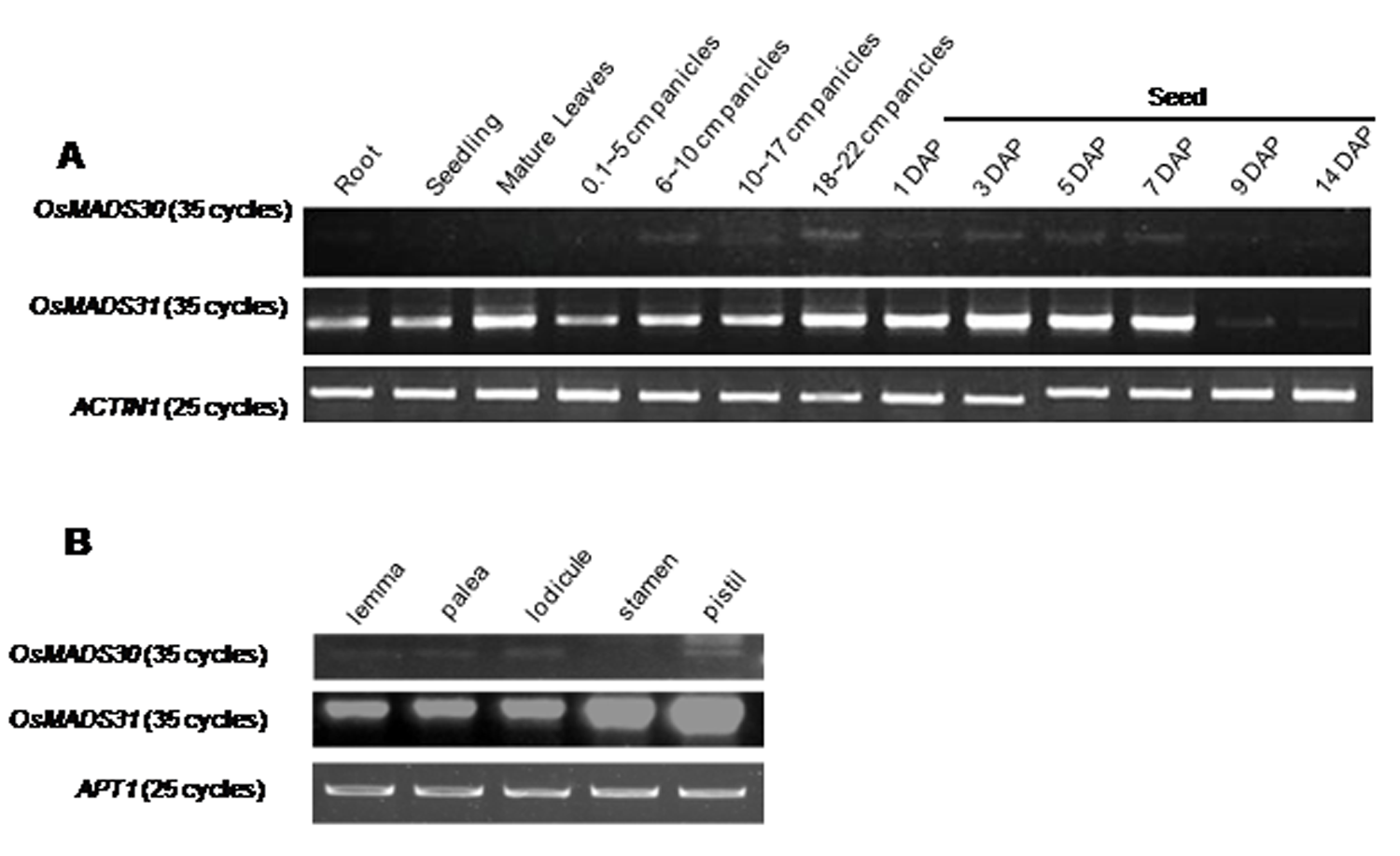

Supplement: Figure S7 — RT-PCR analyses of OsMADS30 and OsMADS31. (A). RT-PCR analyses of OsMADS30 and OsMADS31 at different development stages. DAP, days after pollination. ACTIN1 was used as control. (B). RT-PCR analyses of OsMADS30 and OsMADS31 expression in various floral organs of wild type plants at heading date stage. APT1 was used as a control. (TIF) [file pone.0051435.s007.tif]
