# Supplementary material for: Live and Let Die - The Bsister MADS-Box Gene OsMADS29 Controls the Degeneration of Cells in Maternal Tissues during Seed Development of Rice (Oryza sativa)
Source: PLoS One. 2012 Dec 12;7(12):e51435. doi: 10.1371/journal.pone.0051435 (PMC3520895; doi:10.1371/journal.pone.0051435)
Supplement: Table S1 — Gene specific primers used in this study. (DOCX) [file pone.0051435.s008.docx]

**Text S1. Accession numbers**

Sequence data for this article can be found in the GenBank/EMBL/DDBJ/Phytozome databases under the following accession numbers: *OsMADS2* (L37526), *OsMADS4* (L37527), *OsMADS13* (AF151693), *OsMADS16* (AF077760), *OsMADS29* (AK109522), *OsMADS30* (AY174093), *OsMADS31* (AY177698), *APT1* (AK073627), and *ACTIN1* (AK100267) from rice; *AP3* (M86357), *STK* (NM_001203767), *PI* (D30807), *GOA* (AY141243) and *ABS* (AJ318098) from *Arabidopsis thaliana*; *PtMADS38* (POPTR_0012s14770), *PtMADS45* (POPTR_0015s14950) and *PtMADS30* (POPTR_0007s07620) from *Populus trichocarpa*; *DEFH21* (AJ307056) from *Antirrhinum majus*; *FBP24* (AF335242) from *Petunia hybrida*; *LBS* (AI486443, AI899235) from tomato; *ZMM17* (AJ271208), *ZmBS2* (EB160486) and *ZmBS3* (DR811323) from maize; *WBSis* (AM502893, DQ512369), *TaBS3a* (GH730902), and *TaBs3b* (GH731782) from *Triticum aestivum*; *HBS1* (BQ764751), *HvBS2* (AK373226) and *HBS2* (CK122890) from *Hordeum vulgare*; *BdBS1* (BRADI3G05260 ), *BdBS2* (BRADI1G32210), and *BdBS3* (BRADI5G21700) from *Brachypodium distachyon*; *PvBS1a* (Pavirv00018401m), *PvBS1b* (Pavirv00035216m), *PvBS2* (Pavirv00046240m), *PvBS3a* (Pavirv00008728m) and PvBS3b (Pavirv00020904m) from *Panicum virgatum*; *SiBS1* (Si018183m) and *SiBS2* (Si007187m) from *Setaria italica*; *SbBS1* (XM_002453325), *SbBS2* (XM_002437376) and *SbBS3* (XM_002447045) from *Sorghum bicolor*; *PeBS2* (FP092548) from *Phyllostachys edulis*; *CcBS2* (EB667398) from *Cenchrus ciliaris*; *AaBS* (AY436713) from Aquilegia alpine; *DwBS* (AY436726) from Drimys winteri; *AeAP3*-*2* (AF230698) from *Asarum europaeum*; *GGM13* (AJ132219) from *Gnetum gnemon*.

**Table S1.** Gene specific primers used in this study.

| **Gene name** | **Primer name** | **Sequences (5' to 3')** |
| --- | --- | --- |
| **RT-PCR analysis** | | |
| *OsMADS29* | OsMADS29-F | CACGA TCAGC AAATA TTTGT GG |
|  | OsMADS29-R | ACGAA GGTTG TCCAG CTGCT |
| *OsMADS30* | OsMADS30-F | CAGTG GATGA GCTCA GCCAG |
|  | OsMADS30-R | TCCTA CTGCT TCCAG GAAGT |
| *OsMADS31* | OsMADS31-F | GGTGA TGACT TGGCT TCACT GAC |
|  | OsMADS31-R | TGGTT GCTCA GTTGC ATCCA GAC |
| *APT1* (Control) | APT1-F | ATTCA TTTTT GGTCC GCCC |
|  | APT1-R | CCCAA ATAAC TCATG TGCCT AC |
| *ACTIN1* (Control) | ACTIN1-F | CCAAT CGTGA GAAGA TGACC CA |
|  | ACTIN1-R | CCATC AGGAA GCTCG TAGCT CT |
| **RNAi vecotor construct** | | |
| *OsMADS29* | OsMADS29RNAi-R | GCA GGATCC ACTAGT GGAAG ACCAG AACAG C |
|  | OsMADS29RNAi-F | GGA CCATGG GTTAAC AACAC AGCAA CCC |
| **Quantitative real time RT-PCR** | | |
| *OsMADS29* | qOsMADS29-F | GATGA CTCGG ATGAG GAACG |
|  | qOsMADS29-R | ACGAA GGTTG TCCAG CTGCT |
| *OsAGPS1* | qOsAGPS1-F | GTGCCACTTAAAGGCACCATT |
|  | qOsAGPS1-R | CCCACATTTCAGACACGGTTT |
| *OsAGPS2a* | qOsAGPS2a-F | ACTCCAAGAGCTCGCAGACC |
|  | qOsAGPS2a-R | GCCTGTAGTTGGCACCCAGA |
| *OsAGPL2* | qOsAGPL2-F | AGTTCGATTCAAGACGGATAGC |
|  | qOsAGPL2-R | CGACTTCCACAGGCAGCTTATT |
| *OsAGPL3* | qOsAGPL3-F | AAGCC AGCCA TGACC ATTTG |
|  | qOsAGPL3-R | CACAC GGTAG ATTCA CGAGA CAA |
| *OsVDAC2* | qVDAC2-F | TCACT GTTGC TGGCA CGAAG A |
|  | qVDAC2-R | CTGGT AGGGA AAAGG AATGG ATAG |
| *OsVDAC3* | qVDAC3-F | GGCTC TACAC CGACA TCGGC AAGA |
|  | qVDAC3-R | GCAGC TGTGA TAGTG ACGCC CTCG |
| *OsVPE1* | qVPE1-F | AAGTG GGAGC CGCTG ATTCG |
|  | qVPE1-R | AGAAT CTGGT ACGCA TGGCA C |
| *OsPBZ1* | qPBZ1-F | TGTCC TAAAG TCGGA TGTGC T |
|  | qPBZ1-R | TGCCA TAGTA GCCAT CCACG A |
| *ACTIN1* (Control) | qACTIN1-F | TGCTA TGTAC GTCGC CATCC AG |
|  | qACTIN1-R | AATGA GTAAC CACGC TCCGT CA |
